# Use of horseradish peroxidase for gene-directed enzyme prodrug therapy with paracetamol

**DOI:** 10.1038/sj.bjc.6601780

**Published:** 2004-04-06

**Authors:** J Tupper, G M Tozer, G U Dachs

**Affiliations:** 1 Tumour Microcirculation Group, Gray Cancer Institute, PO Box 100, Mount Vernon Hospital, Northwood, Middlesex, HA6 2JR, UK

**Keywords:** gene therapy, horseradish peroxidase, hypoxia, paracetamol

## Abstract

Gene therapy is a potential method of treating cancer with a greater degree of targeting than conventional therapies. In addition, therapy can be directed towards cells within the tumour population that are traditionally resistant to current treatment schedules. Horseradish peroxidase (HRP) can oxidise paracetamol to *N*-acetyl-*p*-benzoquinoneimine via a one-electron pathway. Incubation of human cells expressing HRP with 0.5–10 mM paracetamol reduced clonogenic survival, but had little effect on control cells. A small increase in apoptosis was seen and a decrease in the number of cells undergoing mitosis, consistent with reports in hepatocytes using higher paracetamol concentrations. The cytotoxicity was also seen under conditions of severe hypoxia (catalyst induced anoxia), indicating that the HRP/paracetamol combination may be suitable for hypoxia-targeted gene therapy.

A number of gene therapy strategies have been proposed for the treatment of cancer. One system is gene-directed enzyme prodrug therapy, or GDEPT. Gene-directed enzyme prodrug therapy is a two-step process which involves the delivery of a gene encoding an enzyme, followed by administration of a nontoxic prodrug, which is converted to a cytotoxin by the enzyme. Several GDEPT combinations have been proposed (Springer and Niculescu-Duvaz, 1996), the most studied being the Herpes Simplex virus thymidine kinase/gancyclovir system (van Dillen *et al*, 2002). This combination has entered clinical trials, and shown safety and some efficacy (Sandmair *et al*, 2000; Miles *et al*, 2001).

Gene-directed enzyme prodrug therapy can be used to selectively target tumour tissue using tissue or environmentally controlled gene expression. In particular, the hypoxic regions of tumours are an attractive target, since severe hypoxia is a tumour-specific condition, and an adverse prognostic factor (Brown and Giaccia, 1998).

The horseradish peroxidase enzyme (HRP) has previously been used to activate indole-3-acetic acid (IAA) and related indoles, as a GDEPT strategy (Greco *et al*, 2000). Horseradish peroxidase catalyses the one-electron oxidation of IAA to the skatole radical, which can undergo a further series of nonenzymatic reactions prior to formation of the cytotoxin (Folkes and Wardman, 2001). The HRP/IAA combination has shown activity under hypoxia (Greco *et al*, 2001), making it suitable for targeting hypoxic regions of tumours. In addition, when transcription of HRP gene expression was placed under the control of hypoxia responsive elements (HREs), the combination showed selective cell kill after hypoxic incubation (Greco *et al*, 2002a).

Paracetamol (acetaminophen) is a widely used analgesic. Its pharmacokinetic and toxicity profiles are widely known and extensively researched. Therefore, it would not require clinical evaluation as a prodrug alone. In cases of overdose, however, the normal elimination pathways become saturated, often due to glutathione (GSH) depletion, and liver damage develops due to the production of *N*-acetyl-*p*-benzoquinoneimine (NAPQI) by cytochrome P450 (CYP) enzymes (Dahlin *et al*, 1984). Alterations in cell cycle progression have also been observed. Horseradish peroxidase has been shown to catalyse the one-electron oxidation of paracetamol to its semiquinone, which can then form polymerisation products, or disproportionate to NAPQI (Potter and Hinson, 1987).

The aim of the current study was to determine whether an HRP/paracetamol combination was capable of producing a cytotoxic species in a GDEPT setting and, in particular, whether it would be suitable for targeting radioresistant hypoxic cells.

## MATERIALS AND METHODS

### Cell culture

Human nasopharyngeal squamous cell carcinoma cells, FaDu, were obtained from the American Type Culture Collection (Manassa, VA, USA). These cells carry a nonsense mutation within the p53 gene (Reiss *et al*, 1992). Cells were maintained in Dulbecco's modified Eagle's medium (DMEM, Life Technologies, Paisley, UK), supplemented with 10% fetal calf serum (Sigma, Poole, UK) and 2 mM L-glutamine (Life Technologies). Cells were kept in a humidified incubator at 37°C and 5% CO_2_/air. Cells were routinely sub-cultured in 75 cm^2^ cantilevered flasks.

Cells were transfected with either the HRP gene (pssHRP-puro, kindly provided by Dr O Greco, Gray Cancer Institute), or the marker green fluorescent protein (GFP, pEGFP-puro) using a nonviral method, as previously described (Greco *et al*, 2000). Stably transfected clones were selected in media containing 1 *μ*g ml^−1^ puromycin (Sigma), which resulted in the death of parental cells within 72 h, and colonies isolated. Gene expression was confirmed by fluorescent-activated cell sorting (FACS) for GFP or HRP enzyme activity, using a modified TMB assay (Greco *et al*, 2000). Horseradish peroxidase activity was seen in HRP and not GFP transfectants. Single clones were isolated, named HRP8 and GFP1, and cell lines derived from these initial clones were used throughout the experiments. Horseradish peroxidase activity expressed as units per mg protein were 0.08±0.003 and 0.002±0.01 for HRP8 and GFP1 cells, respectively. Cells were confirmed as mycoplasma negative using a PCR method (ATCC Mycoplasma Detection Kit Version 2.0).

### Clonogenic assay

Exponentially growing cells were collected from monolayer culture by trypsinisation and plated at low density. Cells were plated as either GFP1 or HRP8, or a mixture of 50% GFP1 and 50% HRP8 cells. Cells were allowed to adhere for 4–6 h. Prodrugs were dissolved in Hanks’ balanced salt solution (HBSS, Life Technologies) and cells were exposed in the 37°C incubator for 4 or 24 h.

Following drug exposure, cells were washed in phosphate-buffered saline (PBS) and grown for approximately 10 days in complete media supplemented with feeder cells (V79 cells exposed to 250 Gy ^60^Co). Colonies were fixed in 0.5% methylene blue w v^−1^ in isomethylated spirit (IMS). Colonies estimated to be greater than 50 cells were counted, and the survival was expressed relative to vehicle-treated controls.

For experiments conducted under anoxic conditions, cells were plated on oxygen impermeable permanox dishes (Nunc), allowed to attach and then moved to an anoxic chamber (Don Whitley Scientific), and media were replaced. After an hour, cells were exposed to prodrug in the chamber. After incubation, medium was removed, cells were washed, and incubated with feeder cells as before. Media and prodrug solutions were kept under anoxia for at least 14 h before addition to cells.

### Glutathione measurements

The GSH level in cells was measured using a commercially available kit (Cayman Chemical, Ann Arbor, MI, USA), following the manufacturer's instructions. Glutathione reacts with Ehlman's reagent to form a yellow coloured 5-thio-2-nitrobenzoic acid (TNB) and a mixed disulphide. The disulphide is reduced by glutathione reductase to recycle the TNB and form further GSH. The rate of TNB production is proportional to the recycling reaction, which is proportional to the concentration of GSH in the sample.

Cells (5 × 10^5^) were collected by scraping and centrifugation. The cell pellet was resuspended and sonicated in phosphate buffer containing 1 mM EDTA. This was centrifuged and the supernatant deproteinated with an equal volume of metaphosphoric acid (MPA, Aldrich, Poole, UK), centrifugation, and treatment with 50 *μ*l of 4 M triethanolamine per millilitre of sample.

Samples were then added to a 96-well plate (TPS), and assay cocktail was added and absorbance at 415 nm was measured every 5 min for 20 min on a Labtech plate reader. GSH levels were calculated by producing a standard curve of known GSH concentrations.

### Cell cycle analysis

Cells were exposed to paracetamol as for clonogenic assays. Following 24 h exposure, cells were washed, and full medium was added for a further 24 h. Cells were then harvested, washed in PBS, fixed in ice-cold 70% ethanol for an hour on ice, rinsed, centrifuged, and the pellet resuspended in a solution containing 20 *μ*g ml^−1^ propidium iodide, 20 *μ*g ml^−1^ RNAse A (Sigma) in PBS. Cells were incubated at 37°C for 30 min, and then analysed by FACS (Becton Dickson, Cowley, Oxford, UK), gated to exclude cellular debris.

### Effect of furafylline on HRP activity

Purified HRP enzyme (0.4 ng, Sigma) was incubated in the presence of varying concentrations of the CYP1A2 inhibitor, furafylline (Sigma), in 80 mM phosphate buffer (pH 5.4). Then, 320 mM 3,3′,5,5′-tetramethylbenzidine (TMB) and 3 mM hydrogen peroxide were added to give a total volume of 2 ml. Absorbance was read every minute at 652 nm for 10 min on a Hewlett Packard model 8452A diode array spectrophotometer. Over this period, absorbance increased linearly.

### Statistical analysis

JMP statistical analysis programme was used to carry out ANOVA and *t*-test analyses.

## RESULTS

**Figure 1:**
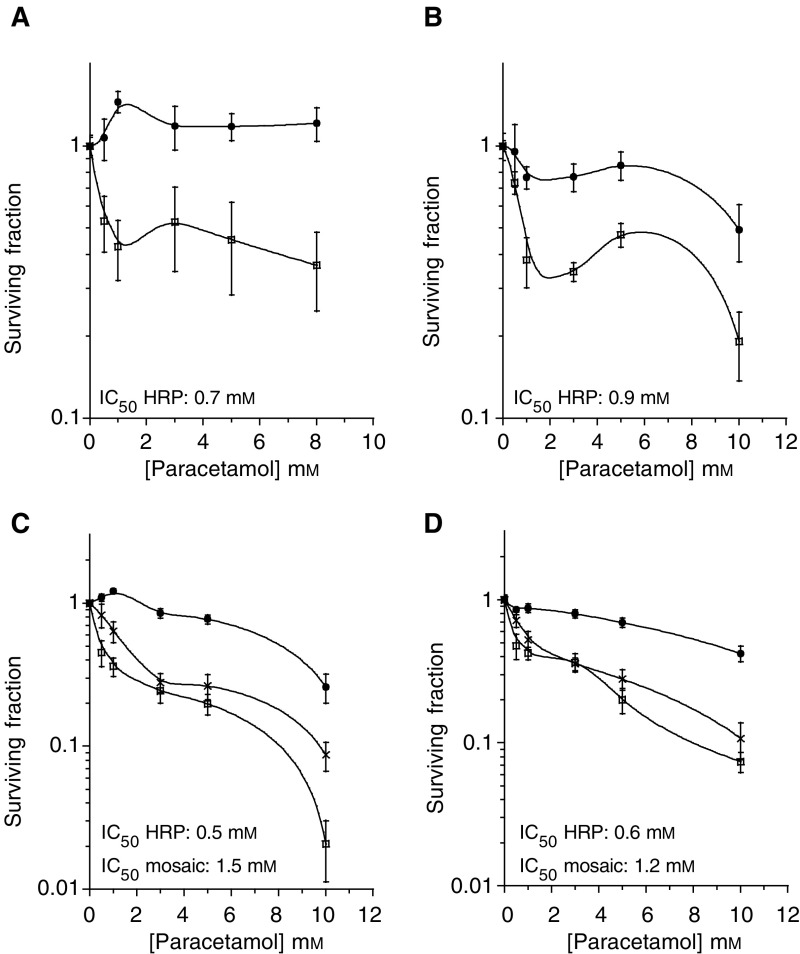
Clonogenic survival of cells following exposure to paracetamol. (**A**) 4 h air, (**B**) 4 h in anoxia (catalyst induced), (**C**) 24 h air, (**D**) 24 h anoxia. Data are mean±s.e.m. three experiments, triplicate samples. • GFP1 controls, □ HRP8 cells, × mixture of 50% HRP8 and 50% GFP1 cells (mosaic).

Incubation with paracetamol for 4 h resulted in a decreased clonogenic ability of FaDu cells stably expressing the HRP enzyme (HRP8). Concentrations of 1 mM prodrug resulted in greater than 50% cell kill (Figure 1A

). Concentrations greater than 1 mM did not appear to further increase the cell kill. There was no loss of viability in GFP controls up to 8 mM paracetamol.

After 24 h exposure to paracetamol, clonogenic survival of HRP8 cells decreased sharply with increasing concentrations of prodrug, with over a log of cell kill at 10 mM paracetamol (Figure 1C). However, the extended incubation resulted in a loss of GFP1 clonogenicity, although to a lesser extent than HRP8.

To determine whether the HRP/paracetamol combination would be suitable for hypoxia targeting, the clonogenic studies were repeated under anoxic conditions. After both 4 and 24 h exposure to paracetamol (Figure 1B and D), HRP8 cells showed a similar decrease in clonogenic survival as under oxic conditions. The IC_50_ values (concentration required to decrease the surviving fraction by 50%) did not differ between the two oxygen status experiments at either exposure time.

Exposure of a mixture of HRP8 and GFP1 cells to paracetamol for 24 h under oxic or anoxic conditions (Figure 1) resulted in cell kill that almost overlapped with that seen in experiments with HRP8 only cells, indicating a large bystander effect.

**Figure 2:**
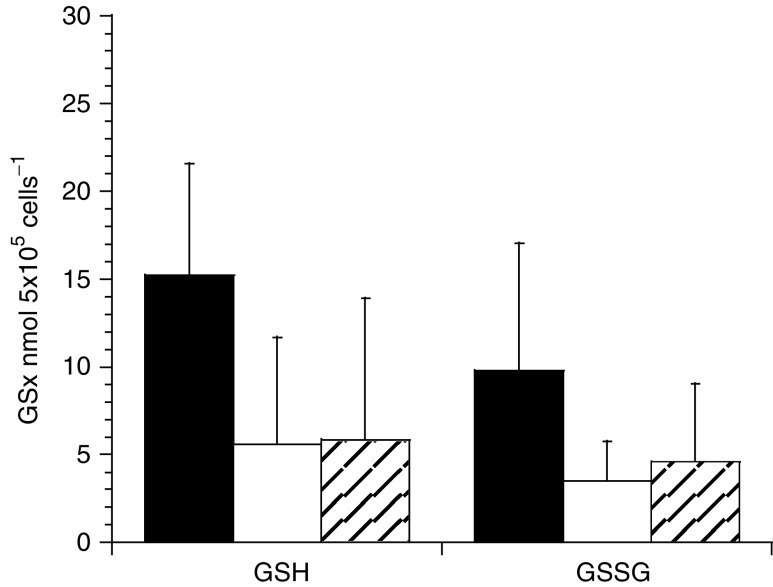
Glutathione concentrations in FaDu cells measured using the recycling assay. ▪ FaDu parental cells, □ GFP1 clones, ▪ HRP8 clones. Data are mean±s.d. two experiments, duplicate samples.

There was no significant difference in either the oxidised or reduced form of GSH between HRP8 or GFP1 cells as determined by the recycling assay (Figure 2

). There was a slight but not significant difference between GSx levels in parental FaDu cells and HRP8 and GFP1 clones.

**Figure 3:**
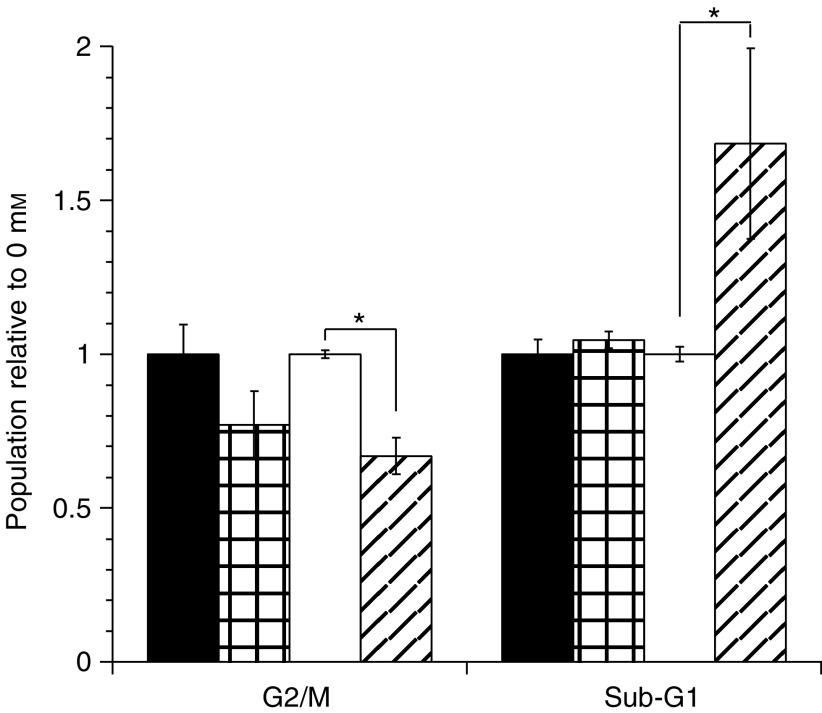
Effect of paracetamol on cell cycle. Cells were exposed to paracetamol in air for 24 h followed by 24 h recovery before fixation, staining with propidium iodide, and FACS analysis. Data are mean±s.e.m. three experiments, duplicate samples. ▪ GFP 0 mM, ⊞ GFP 1 mM paracetamol, □ HRP 0 mM, ▪ HRP 1 mM paracetamol. ^*^<0.05 *t*-test and ANOVA.

Propidium iodide staining of cells showed a significant increase in the sub-G_1_ cell population of HRP8 cells following incubation with paracetamol compared with untreated controls (Figure 3

). There was also a small decrease in the number of G_2_/S cells indicating a decrease in proliferation. In contrast, there was no statistically significant change in the cycle distribution of GFP1 cells.

**Figure 4:**
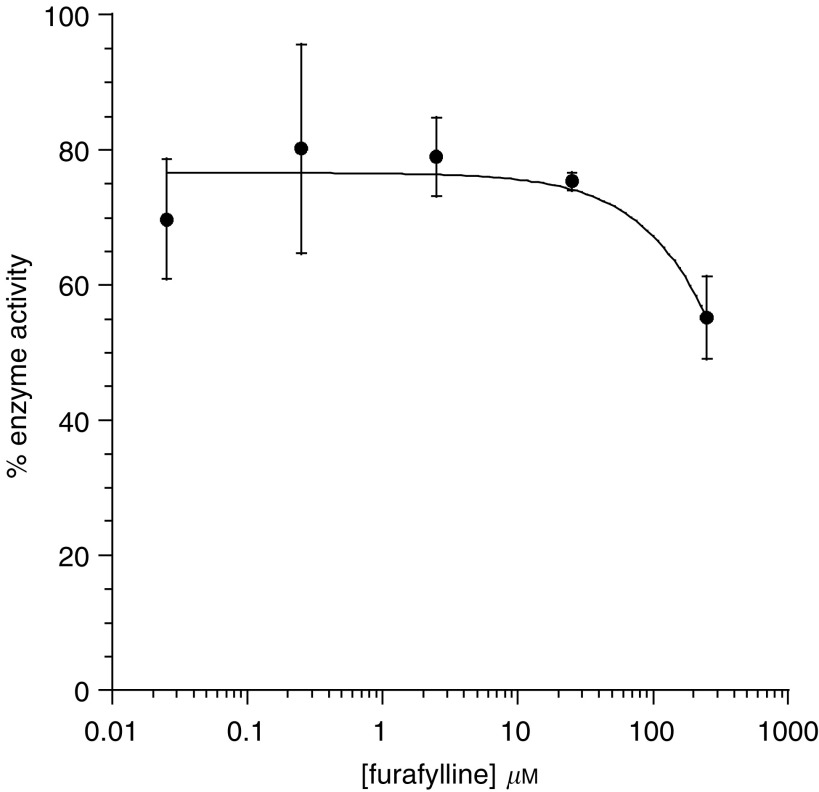
Effect of furafylline on HRP enzyme activity. Purified enzyme was incubated with furafylline in buffer solution for 10 min prior to assay. Data are expressed as percentage of control activity, mean±s.d. triplicate samples.

In experiments using purified HRP enzyme, incubation with up to 25 *μ*M furafylline (a potent cytochrome P450 1A2 inhibitor) led to a maximum decrease of 20% in the enzyme activity (Figure 4

). Concentrations of furafylline required to decrease the enzymatic activity of purified HRP enzyme by half were not attainable as the solubility limit of the drug was reached at 250 *μ*M.

## DISCUSSION

The results presented here show the ability of the HRP enzyme to catalyse the conversion of paracetamol to a cytotoxin intracellularly. This enzyme/prodrug combination was effective under both oxic and anoxic conditions, with no loss of activity in the absence of oxygen. In addition, mixtures of HRP-expressing and nonexpressing cells showed almost equivalent levels of cell kill to HRP only experiments, indicating a large bystander effect.

The potential of delivering the HRP gene to activate IAA has been shown previously (Greco *et al*, 2000). In the present study, paracetamol was used as an alternative prodrug to indoles. Sufficient cytotoxin was produced to decrease the clonogenic ability of HRP transfectants both under oxic and anoxic conditions. This effect appears to be independent of p53, since FaDu cells carry a nonsense mutation at codon 248 (Reiss *et al*, 1992).

The toxicity of the HRP/paracetamol combination is likely to be a result of NAPQI formation. However, the exact mechanism is currently unclear. The data showed a slight increase in apoptosis, as indicated by an increased sub-G_1_ peak of treated cells after propidium iodide staining, and also a decrease in the number of cells undergoing mitosis (increased G2/M population). However, this may be supplemented by necrosis, since this is the primary finding *in vivo* following overdose (Timbrell, 1996), and the decrease in clonogenicity seen following paracetamol exposure could not be fully explained by apoptosis. Further experiments are required to determine the exact route of cell death and mechanism of action.

Paracetamol/NAPQI is able to deplete cellular GSH levels (Adamson and Harman, 1993; Pumford *et al*, 1997), bind to proteins and DNA (Pumford *et al*, 1997), increase intracellular calcium concentrations (Tsokos-Kuhn, 1989), cause lipid peroxidation, increase reactive oxygen species (Manov *et al*, 2002), and has been shown to affect cell cycle progression and DNA synthesis (Djordjevic *et al*, 1986; Richard *et al*, 1991). There is some controversy over which mechanism is most important, and its, likely that all play a role, with different mechanisms predominating under differing conditions. These effects, described in the literature, were seen in cells where NAPQI production would be due to a direct two-electron oxidation by CYP enzymes. In the case of activation by HRP via one-electron oxygenation, it is possible that there could be constant recycling of the semiquinone back to paracetamol with little formation of NAPQI, provided there is sufficient GSH or NADPH (preferentially GSH (Potter and Hinson, 1987)). This in itself would lead to oxidative stress, and may increase the susceptibility of cells to damage by ionising radiation. Radiotherapy is a standard treatment for many solid tumours, and its combination with GDEPT strategies is promising (Buchsbaum *et al*, 1996). In fact, the HRP/IAA combination sensitised cells to radiation in air and hypoxia (Greco *et al*, 2002b). Hence, future work is aimed at determining whether the HRP/paracetamol combination would act as radiosensitizer.

There was no detectable difference in GSH levels between GFP1 and HRP8 cells, indicating that the increased susceptibility of HRP8 transfectants to paracetamol is unlikely to be due to an exacerbation of an imbalanced oxidative state. It has previously been reported that the addition of HRP to cells can decrease GSH levels (Harman *et al*, 1986). From our results it appears that the constant production of the enzyme intracellularly has no greater effect on GSH concentrations than the production and persistence of GFP. Overall, however, transfected cells tended to have lower GSH levels than untransfected FaDu cells. This could be due to either the presence of the transgene, or may be a consequence of the presence of puromycin antibiotic in the growth media.

The concentrations of paracetamol used to achieve cell kill are similar to those used by Thatcher *et al* (2000) for prodrug activation using CYP1A2. This group showed decreased cell viability following exposure of fibroblasts overexpressing CYP1A2 to paracetamol. The levels used in both studies are greater than those achievable after current therapeutic doses of paracetamol. However, it may be possible to increase paracetamol doses in patients by combining treatment with furafylline, a potent CYP1A2 inhibitor (Sesardic *et al*, 1990). Plasma steady-state levels of furafylline can reach 5.8 *μ*M in humans (Tarrus *et al*, 1987), and the IC_50_ for purified CYP1A2 is 0.07 *μ*M (Sesardic *et al*, 1990), indicating that the use of furafylline to inhibit paracetamol activation in the liver is feasible. Importantly for our study, furafylline had little effect on HRP enzyme activity up to 25 *μ*M. Orally administered methionine or *N*-acetyl cysteine could also be administered to increase liver GSH levels (McLean and Day, 1975; Aebi and Lauterburg, 1992). This would decrease the susceptibility of the liver to damage by NAPQI by allowing increased amounts of mercapturic acid to be formed, as well as *N*-acetylcysteine conjugates. Although somewhat effective at minimising damage following paracetamol overdose, the effect of GSH precursors administered prior to paracetamol in combination with furafylline needs to be assessed.

In conclusion, this data demonstrates for the first time the potential for HRP/paracetamol as a GDEPT strategy, under tumour conditions. Further work needs to be carried out to determine the mechanism of action, the *in vivo* potential and possible radiosensitising effects before speculating on clinical trial outcome.
